# Transcription Factor *GarWRKY5* Is Involved in Salt Stress Response in Diploid Cotton Species (*Gossypium aridum* L.)

**DOI:** 10.3390/ijms20215244

**Published:** 2019-10-23

**Authors:** Qi Guo, Liang Zhao, Xinqi Fan, Peng Xu, Zhenzhen Xu, Xianggui Zhang, Shan Meng, Xinlian Shen

**Affiliations:** 1 Key Laboratory of Cotton and Canola Research at the Lower Reach of the Yangtze River Plain, Ministry of Agriculture and Rural Affairs, Nanjing 210014, China; sevenguo86@163.com (Q.G.); liangz@jaas.ac.cn (L.Z.); fxq861223@aliyun.com (X.F.); semon528@hotmail.com (P.X.); lele20032@163.com (Z.X.); mshan84@163.com (S.M.); 2 The Institute of Industrial Crops, Jiangsu Academy of Agricultural Sciences, Nanjing 210014, China; 3 Institute of Life Sciences, Jiangsu University, Zhenjiang 212013, China

**Keywords:** expression patterns, *Gossypium aridum*, RNA-Seq, salt stress, virus-induced gene silencing (VIGS), WRKY

## Abstract

Cotton is one of the most economically important crops in the world, and it is exposed to various abiotic stresses during its lifecycle, especially salt stress. However, the molecular mechanisms underlying cotton tolerance to salt stress are still not fully understood due to the complex nature of salt response. Therefore, identification of salt stress tolerance-related functional genes will help us understand key components involved in stress response and provide valuable genes for improving salt stress tolerance via genetic engineering in cotton. In the present study, virus-induced gene silencing of *GhWRKY5* in cotton showed enhanced salt sensitivity compared to wild-type plants under salt stress. Overexpression of *GarWRKY5* in *Arabidopsis* positively regulated salt tolerance at the stages of seed germination and vegetative growth. Additionally, *GarWRKY5*-overexpressing plants exhibited higher activities of superoxide dismutase (SOD) and peroxidase (POD) under salt stress. The transcriptome sequencing analysis of transgenic *Arabidopsis* plants and wild-type plants revealed that there was enriched coexpression of genes involved in reactive oxygen species (ROS) scavenging (including glutamine S-transferases (GSTs) and SODs) and altered response to jasmonic acid and salicylic acid in the *GarWRKY5*-OE lines. *GarWRKY5* is involved in salt stress response by the jasmonic acid- or salicylic acid-mediated signaling pathway based on overexpression of *GarWRKY5* in *Arabidopsis* and virus-induced gene silencing of *GarWRKY5* in cotton.

## 1. Introduction

WRKY proteins comprise one of the largest transcription factor families in plants. The conserved WRKY domain contains approximately 60 amino acid residues. The WRKY domain is defined based on the conserved WRKYGQK hexapeptide sequence and is usually followed by a C2H2- or C2HC-type zinc finger motif at the N-terminal end. WRKY transcription factors are classified on the basis of both the number of WRKY domains and the zinc finger motifs that they contain; WRKY proteins with two WRKY domains belong to group I, whereas Group II and Group III members have only a single WRKY domain, followed by a novel zinc finger-like motif C2H2 (C–X4-5–C–X22–23–H–X–H) and C2HC (C–X7–CX23–H–X–C), respectively [1]. WRKY proteins play diverse roles in regulating plant defense responses and developmental and physiological processes of plants. In addition to their role in plant development, WRKY family genes are also important in regulating plant biotic and abiotic stresses, for example, pathogen-induced defense pathways, drought, salt stress, and others [2,3,4]. An increasing number of studies has been reporting that WRKY genes are involved in regulating plant responses to salt stress. Their function has been elucidated using genetic and molecular approaches in different species, such as *AtWRKY25* and *AtWRKY33* in *Arabidopsis* [5], *OsWRKY11* and *OsWRKY45* in rice [2], *GmWRKY13* and *GmWRKY54* in soybean [6], and *TaWRKY10* in wheat [7].

Cotton is one of the most economically important crops in the world, and it endures various abiotic stresses during its lifecycle, especially salt stress. However, the molecular mechanisms underlying cotton tolerance to salt stress are still not fully understood due to the complex nature of this response. With the release of the *Gossypium* genome sequence [8,9,10], genome-wide identification of WRKY family genes has been conducted in *Gossypium raimondii*, *Gossypium arboreum* and *Gossypium aridum* [11,12,13,14]. Several studies have suggested the importance of specific WRKYs in the transcriptional regulation of salt-related genes in cotton. For example, overexpression of *GhWRKY25* from *Gossypium hirsutum* in *Nicotiana benthamiana* enhanced tolerance to salt stress [15], and *GhWRKY39-1*-overexpressing plants exhibited increased tolerance to salt and oxidative stress and increased transcription of antioxidant enzyme genes [16]. Overexpression of *GhWRKY34* in *Arabidopsis* resulted in a transgenic plant with increased tolerance to salt stress [17]. The ectopic expression of the *GhWRKY6*-like gene significantly increased salt tolerance in *Arabidopsis thaliana*, while silencing of the *GhWRKY6*-like gene increased the sensitivity of cotton to abiotic stresses [18]. Despite the abovementioned reports, the molecular mechanisms by which WRKY transcription factors (TFs) regulate salt stress still remain largely unclear in cotton.

In a previous study, 109 *GarWRKY5* genes were identified in a salt-tolerant wild cotton species, *Gossypium aridum*, based on transcriptome sequencing data. Meanwhile, 28 salt-responsive *GarWRKY* genes were identified from transcriptome data and real-time quantitative PCR analysis [14]. Of the cotton WRKY genes, *GarWRKY5* belongs to Group III of the WRKY family. The open reading frame of *GarWRKY5* (GenBank accession number KM438453) is 921 bp, and it putatively encodes a 306-amino acid protein. In the roots of G. *aridum*, *GarWRKY5* genes were activated within 3 h of salt treatment, with expression peak at 72 h. This observation implies a potentially important role for *GarWRKY5* in mediating NaCl stress responses. In the present study, we further confirmed the role of *GarWRKY5* in salt stress response by overexpression of *GarWRKY5* in *Arabidopsis* and by silencing it in upland cotton. The expression profile of *GarWRKY5*-OE lines and wild-type (WT) plants was measured to investigate the possible mechanisms by which *GarWRKY5* participates in salt stress responses.

## 2. Results

### 2.1. Characterization of GarWRKY5 Based on Structure, Evolution, and Expression

**Figure 1:**
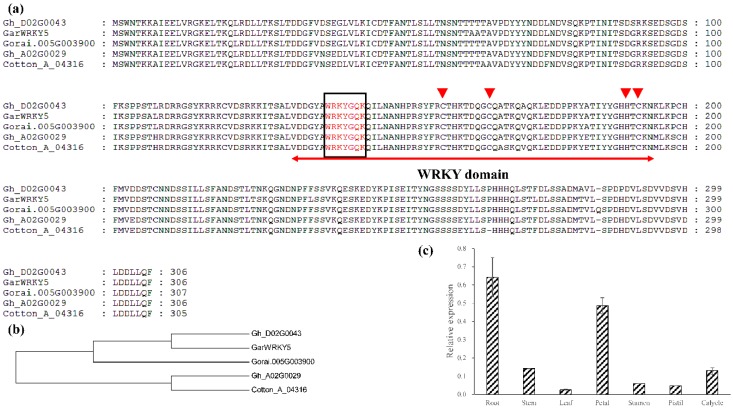
Structure, evolution, and expression of *GarWRKY5.* (**a**) Identification of the WRKY domain. The approximately 60-amino acid WRKY domain and C–X_7_–CX_23_–HXC-type zinc finger-like motif. The 60 amino acid WRKY domain was indicated by a red line and the conserved WRKYGQK sequence was black squares. The C and H residues of zinc finger domain were indicated by red triangles. (**b**) Phylogenetic analysis of *GarWRKY5* between homologous genes from *G. hirsutum* (Gh_A02G0029/Gh_D02G0043), *G. raimondii* (Gorai.005G003900), and *G. arboreum* (Cotton_A_04316). (**c**) The expression of *GarWRKY5* in different tissues and organs from *G. aridum*. The relative expression levels were calculated with three biological replicates and three experimental replicates. Standard deviation (SD) was used to calculate the error bars.

Based on previous studies, *GarWRKY5* encodes a member of Group III of WRKY. The predicted GarWRKY5 proteins and homologous genes from *G. hirsutum* (Gh_A02G0029/Gh_D02G0043), *G. raimondii* (Gorai.005G003900), and *G. arboreum* (Cotton_A_04316) contain an approximately 60-amino acid WRKY domain that is composed of the conserved amino acid sequence (WRKYGQK) and a zinc finger motif (C–X_4-5_–CX_22-23_–H–X_1_–C) (Figure 1a). Based on the evolutionary tree, Gorai.005G003900 from the G. *raimondii* genome was close to Gh_D02G0043 from the Dt-subgenome of allotetraploid cotton in evolutionary relationships. The paralogous pairs had ratios of nonsynonymous to synonymous substitutions (Ka/Ks) of more than 1.0 between *GarWRKY5* and the other four genes except Gh_D02G0043, indicating that they had gone through positive selection in the evolutionary process (Figure 1b, Table S1). In terms of expression in vegetative and reproductive organs of G. *aridum*, *GarWRKY5* had a higher expression level in the root than in other organs (Figure 1c).

### 2.2. Silencing GarWRKY5 in Upland Cotton Line Compromises Salt Tolerance

**Figure 2:**
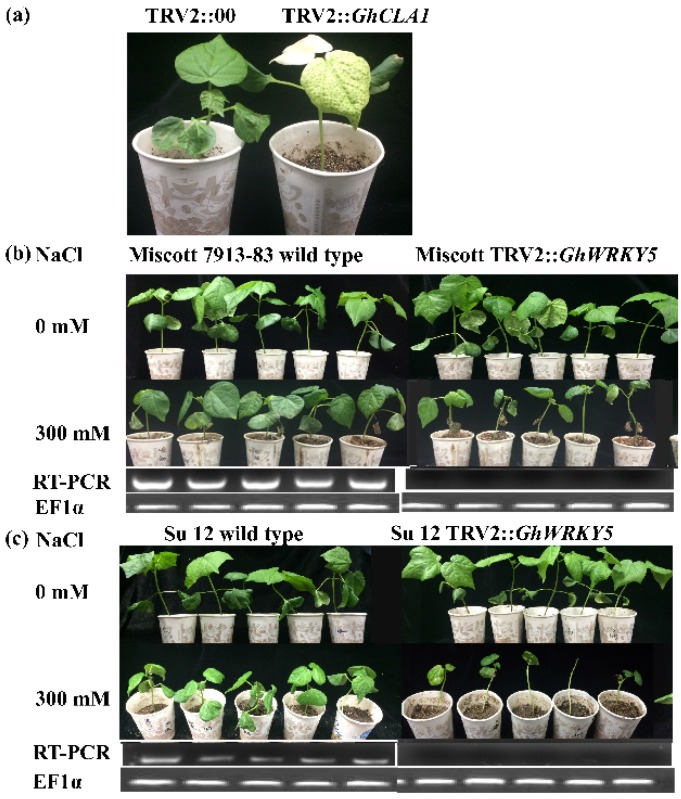
The identification of salt tolerance in the *GhWRKY5*-silenced plants by virus-induced gene silencing (VIGS). (**a**) The leaves of TM-1 turned white after TRV2::*GhCLA1* gene silencing, and empty vector (TRV2:00) leaves remained as green as the wild-type (WT) TM-1. (**b**) The leaves of “Miscott”, a salt-tolerant cultivar, withered, and new leaves grew slowly. (**c**) The leaves of “Su12”, a salt-sensitive cultivar, withered, fell off, and new leaves grew slowly. VIGS experiments were repeated at least three times with more than 10 plants for each construct per repeat.

To elucidate the role of *GarWRKY5*, the virus-induced gene silencing (VIGS) method was used to knockdown the expression of *GhWRKY5*, a homologous gene of *GarWRKY5* in upland cotton. After growing plants in an illumination incubator for one week, we hand-infiltrated *Agrobacterium* cultures carrying the VIGS vector into cotton cotyledons. Approximately 7 d after agroinfiltration, leaves of the *GhCLA1*-silenced plant displayed the photobleaching phenotype as expected, which was uniformly distributed on the entire true leaves (Figure 2a), suggesting that the VIGS system can work well based on our experimental conditions. To investigate the silencing efficiency of *GhWRKY5* in the tested plants, semiquantitative RT-PCR was used to determine the expression levels. The results showed that the *GhWRKY5* expression level in the silenced plants was much lower than in the control plants. At least five *GhWRKY5*-silenced plants with four true leaves were treated with 300 mM NaCl solution; distilled deionized water was used as the control. Ten days later, the tolerance of the TRV::*GhWRKY5* plants decreased significantly compared to TRV::00 (infiltrated with empty vector) plants in two upland cultivars, with the growth of TRV::*GhWRKY5* plants from the salt-sensitive cultivar (Su12) being inhibited more severely than that of TRV::*GhWRKY5* plants from the salt-tolerant cultivar (Miscott 7913-83) (Figure 2b,c). VIGS experiments were repeated at least three times with more than 10 plants for each construct per repeat.

### 2.3. Overexpression of GarWRKY5 Regulates Salt Tolerance in Arabidopsis

To further analyze the function of *GarWRKY5* under salt stress conditions, we generated *GarWRKY5*-overexpressing lines in *Arabidopsis* for phenotypic observation and physiological analysis. Three positive transgenic *Arabidopsis* lines (lines 1, 6, and 14) with high expression levels of *GarWRKY5* were selected for further analysis.

**Figure 3:**
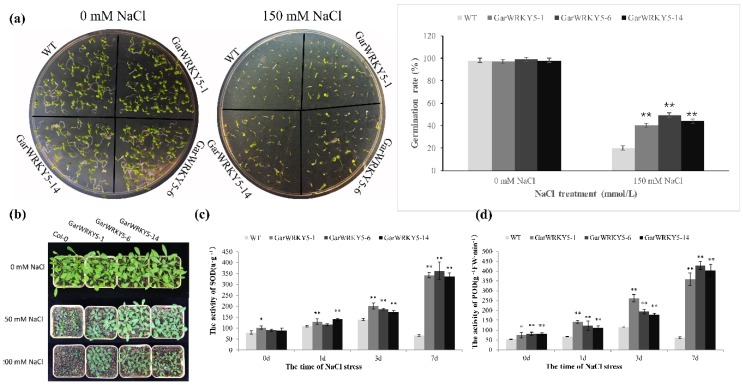
Overexpression of *GarWRKY5* regulates salt tolerance in *Arabidopsis.* (**a**) The germination rate of transgenic *Arabidopsis* lines overexpressing *GarWRKY5* gene. (**b**) *GarWRKY5*-OE lines were grown on soil medium containing 0, 150, and 200 mM NaCl. (**c**) The activity of superoxide dismutase (SOD). (**d**) The activity of peroxidase (POD). The SOD and POD activities were assayed in the three independent *GarWRKY5*-overexpressed transgenic lines (*GarWRKY5*-1, *GarWRKY5*-6, and *GarWRKY5*-14), with three independent biological replicates of each line. Student’s *t*-test: ^*^p < 0.05 and ^**^p < 0.01. SD was used to calculate the error bars.

The transgenic *Arabidopsis* lines overexpressing the *GarWRKY5* gene were germinated on solid medium containing 0 or 150 mM NaCl. The germination rates and root length showed no significant difference between WT and transgenic plants under normal growth conditions. However, the germination rates of the three *GarWRKY5*-overexpressing lines (*GarWRKY5*-1, *GarWRKY5*-6, and *GarWRKY5*-14) were significantly improved compared to the WT (40.0%, 49.3%, and 44.0% vs. 20.0%, respectively) (Figure 3a). The 20-day-old *GarWRKY5*-overexpressing transgenic plants were treated with 150 mM or 200 mM NaCl solution, with the distilled deionized water used as the control. Four weeks later, growth of both the *GarWRKY5*-overexpressing transgenic and the WT plants was significantly inhibited, while the *GarWRKY5*-overexpressing seedlings remained green and continued to grow (Figure 3b). These data indicated that *GarWRKY5* positively regulated salt tolerance at the stage of seed germination and vegetative growth.

To study the physiological response of *GarWRKY5*-overexpressing *Arabidopsis* plants to salt stress, the three transgenic lines (*GarWRKY5*-1, *GarWRKY5*-6, and *GarWRKY5*-14) were selected to further analyze the activities of superoxide dismutase (SOD) and peroxidase (POD) in leaves of WT and transgenic plants. The SOD activity of transgenic plants was significantly higher than that of WT plants with or without salt stress. In particular, the SOD activity of the three transgenic lines, relative to the wild-type plants under salt stress, increased 5.1 times (*GarWRKY5*-1), 5.5 times (*GarWRKY5*-6), and 5.0 times (*GarWRKY5*-14) in the seventh day and 1.2 to 1.4 times in the first and third day (Figure 3c). The POD activity of transgenic plants was significantly improved in the WT plants under salt stress. The activity of POD in the three transgenic lines increased 5.8 times (*GarWRKY5*-1), 7.0 times (*GarWRKY5*-6), and 6.6 times (*GarWRKY5*-14) in the seventh day and 1.6 to 2.3 times in the first and third day (Figure 3d). Plants have complex antioxidative defense systems to maintain reactive oxygen species (ROS)-scavenging ability and control intracellular homeostasis [19,20]. These data demonstrated that overexpression of *GarWRKY5* in transgenic *Arabidopsis* plants resulted in increased activity of antioxidative enzymes, which was associated with the increased salt tolerance of the transgenic *Arabidopsis* plants.

### 2.4. The GarWRKY5 Regulatory Network in Salt Stress

To identify potential target genes of *GarWRKY5*, we then performed RNA sequencing (RNA-Seq) analysis of 35S:*GarWRKY5* and wild-type plants grown under salt stress for 0 or 3 d. In total, the expression of 398 genes was significantly changed in the *GarWRKY5*-OE lines compared to the wild-type plants under normal growth conditions (0 d NaCl). Among them, 253 differentially expressed genes (DEGs) were upregulated in the OE lines, whereas 145 DEGs were downregulated (OE 0 d vs. WT 0 d; Table S2). These genes represented the candidate downstream genes regulated directly or indirectly by *GarWRKY5*.

GO enrichment analysis was performed on the 253 upregulated DEGs and 145 downregulated genes, respectively. For the upregulated DEGs, under “biological process,” oxidant detoxification, response to jasmonic acid (JA) and salicylic acid (SA), response to salt stress and osmotic stress were significantly enriched, like glucosyltransferase activity and calcium ion binding in “molecular function,” and vacuole and protein-containing complex in “cellular component.” For downregulated DEGs, response to ethylene, oxidative stress and abiotic stimulus were significantly enriched in “biological process,” but no categories were significantly enriched with respect to “molecular function” and “cellular component” (Table S3).

Gene Ontology (GO) enrichment analysis showed there were 19 DEGs involved in salt stress and the osmotic stress process. These 19 genes were grouped into two main categories, namely, “ROS scavenging” and “response to hormone”. The “ROS-scavenging” group included glutathione S-transferases (GSTs) (AT1G02920 and AT1G02930) and SOD (AT1G08830 and AT2G28190). The “response of hormone” group included jasmonic acid response genes (AT1G43160, AT1G56650, AT3G16470, AT4G23600, and AT5G24770) and salicylic acid response genes (AT1G43160 and AT2G33380) (Table 1).

Because *GarWRKY5* is homologous to *AtWRKY70* [14], we performed network analysis for these 19 DEGs and *AtWRKY70* using the STRING database, version 11.0 (https://string-db.org/). The results showed that *AtWRKY70* could regulate AT1G02930 (glutathione S-transferase F6, GSTF6) and AT5G24770 (acid phosphatase VSP2) (Figure S1). In addition, we analyzed the promoters (1-kb upstream of the translation start sites) of the 19 DEGs using the JASPAR database [21]. Promoter region screening showed that all 19 DEGs had 3–10 W-box motifs in their promoter regions, a DNA-sequence motif (T)TGAC(C/T) that could bind to a WRKY transcription factor (Table 1). Based on the data presented in this study, we hypothesize that *GarWRKY5* may be a positive transcription regulator involved in plant response to high-salinity stress through the ROS-scavenging system, such as by activating expression of GST and SOD genes by jasmonic acid-mediated or salicylic acid-mediated signaling pathway.

## 3. Discussion

WRKY TFs are key regulators of many plant processes, including responses to biotic and abiotic stresses, senescence, seed dormancy, and seed germination [22]. Recent studies have broadened our knowledge of the WRKY TF family and its functions in salt stress responses in cultivated cotton [18,23,24]. However, wild relatives of crops represent potentially valuable gene pools and are the primary source of important genes. In a previous study [14], using a D-genome diploid species (*G. aridum*) from the Pacific coastal states of Mexico, which shows remarkable tolerance to salt stress, we set out to perform transcriptome analysis and identified the response of 28 WRKY TFs in *G. aridum* to salt stress conditions. Based on overexpression of *GarWRKY17* and *GarWRKY104* in *Arabidopsis*, functional analysis indicated that these two genes could positively regulate salt tolerance in different developmental stages of transgenic *Arabidopsis* [14]. In the present study, we have provided evidence that *GarWRKY5* positively regulates salt stress by overexpressing *GarWRKY5* in *Arabidopsis* and silencing it in upland cotton. Together with the findings from the previous study, we conclude that *GarWRKY5* from *G. aridum* may play a significant role in modulation of salt stress response and can potentially be utilized to improve salt tolerance in cultivated cotton and other crops.

In salt stress, plants can accumulate ROS and enhance the expression of ROS-scavenging enzymes. Alleviation of oxidative damage by scavenging ROS is an important strategy by which plants can tolerate stress [25]. Transgenic plants overexpressing ROS-scavenging enzymes, such as SOD, GST/glutathione peroxidase (GPX), and ascorbate peroxidase (APX), have shown increased tolerance to osmotic stress, oxidative stress, and temperature [26,27,28]. In the current study, the activities of POD and SOD were higher in the *GarWRKY5*-OE lines than in the wild-type plants and contributed to the increased salt tolerance of the transgenic *Arabidopsis* plants. Meanwhile, based on GO enrichment and prediction of the W-box motif, ROS-scavenging genes were enriched, including GSTs (AT1G02920 and AT1G02930) and SODs (AT1G08830 and AT2G28190), in the *GarWRKY5*-OE lines. They contained 6–10 W-box motifs at their promoter region. GSTs and SODs could be regarded as candidate target genes to which *GarWRKY5* binds. Taken together, we hypothesize that *GarWRKY5* may be a positive transcription regulator in response to high salinity stress through the ROS-scavenging system by activating expression of GST genes, highlighting the importance of *GarWRKY5* as a metabolic engineering tool for improvement of salt stress in cotton.

*GarWRKY5* shows sequence homology with *OsWRKY45* in rice and *AtWRKY70* in *Arabidopsis* [14]. The expression of rice *WRKY45* (*OsWRKY45*) was markedly induced in response to the stress-related hormone abscisic acid (ABA) and various stress factors, e.g., application of NaCl, polyethylene glycol (PEG), mannitol, or dehydration. Constitutive overexpression of *OsWRKY45* conferred a number of properties to transgenic plants, including increased resistance to bacterial pathogen, and increased tolerance to salt and drought stresses in *Arabidopsis* [29]. GST and cytochrome P450 genes are regulated by *WRKY45* in rice [30]. In this study, network analysis for *AtWRKY70* and the 19 DEGs enriched with respect to the salt stress and osmotic stress processes by the STRING database showed that *AtWRKY70* could regulate AT1G02930 (GSTF6). All these observations show that *GarWRKY5* may have regulatory mechanisms similar to those of *OsWRKY45* in rice and *AtWRKY70* in *Arabidopsis*.

Plant annexins are thought to be signals that link ROS- and [Ca^2+^] cyt-driven [31,32] and could be involved in H_2_O_2_-activated Ca^2+^ fluxes. These soluble proteins have the capacity for membrane association or insertion [33]. The *Arabidopsis* annexin genes are differentially regulated by exposure to salt, drought, and high- and low-temperature conditions. In NaCl treatment, *AnnAt4* (AT2G38750) responded strongly at the transcriptional level and showed a very high rate (383-fold) compared to control [34]. Our results demonstrate that the promoter of AT2G38750 could identify WRKY TF binding site. Further work is still needed to confirm the relationship between *GarWRKY5* and *AnnAt4* (AT2G38750) by experiment.

Previous studies have demonstrated that Group III WRKY members may play prominent roles under biotic and abiotic stress responses. For example, overexpression of a grape Group III WRKY transcription factor gene, *VlWRKY48*, in *A. thaliana* increased disease resistance and drought stress tolerance [35]. Another Group III member, *AtWRKY46*, functioned in both basal resistance against pathogens and tolerance to oxidative stress and aluminum toxicity induced by drought, salt, and oxidative stresses [36]. Overexpressed *OsWRKY45* in *Arabidopsis* increased pathogen defense, drought, and salt resistance [29]. Overexpression of *AtWRKY70* led to upregulation of PR genes and downregulation of PDF1.2, leading to enhanced resistance against biotrophic pathogens and enhanced susceptibility to necrotrophic pathogens. *AtWRKY70,* as a repressor of JA-responsive genes and an activator of SA-induced genes, integrating signals from these mutually antagonistic pathways [37]. The function of Group III WRKY members may be a node of convergence that integrates biotic and abiotic stress signals, so they have great potential for increased stress tolerance [38]. Encoding a member of the Group III WRKY family, the potential role of *GarWRKY5* in mediating response to multiple stress factors needs to be further investigated.

Based on the data presented in this study, *GarWRKY5* exhibited significant salt tolerance function by virus-induced gene silencing strategy in cotton and overexpressing it in *Arabidopsis*. Enhanced tolerance to salt stress may be due to the ROS-scavenging system, such as activating expression of GST and SOD genes by the jasmonic acid- or salicylic acid-mediated signaling pathway. These results suggest that *GarWRKY5* functions as a transcription factor and plays an important role in improving salt tolerance. Further elucidation of the molecular mechanism of *GarWRKY5* will require genetic transformation in cotton. It will provide novel insights into the complicated regulatory network controlling salt tolerance in cotton.

## 4. Materials and Methods

### 4.1. Plant Materials and Treatment Conditions

The National Wild Cotton Plantation in Hainan Island, China, kindly supplied seeds from the wild *Gossypium* species G. *aridum*. The same treatment procedure was used as described by Xu et al. (2013) [39]. The G. *aridum* seeds were germinated in distilled deionized water. The growth conditions were 60% humidity, day and night temperatures of 28 °C and 23 °C, respectively, and photoperiod of 12 h light/12 h dark in the growth chamber. The germinated seeds were planted into nutritional soil and cultured in the plant growth chamber with the same set conditions. Uniform cotton seedlings measuring about 20 cm in height and with four true leaves were transferred into paper cups with 1× Hoagland’s nutrient solution. After three days, the uniform cotton seedlings were treated with 200 mM NaCl for 0, 1, 3, 6, 12, 24, and 72 h, and untreated seedlings were used as the control. Root and leaf tissues were collected at each stage under salt stress treatment. All samples were immediately frozen in liquid nitrogen and stored at −70 °C.

### 4.2. Phylogenetic Analysis of GarWRKY5 Genes

A phylogenetic tree was constructed as follows. First, the ClustalW software was used to sequence alignment. Then, the phylogenetic tree was constructed by the maximum likelihood (ML) method in MEGA 7.0 software [40] (https://www.megasoftware.net/) with 1000 replicates bootstrap test. The ratios of nonsynonymous to synonymous substitutions (Ka/Ks) between the paralogous pairs were analyzed based on DnaSP v6.0 software [41].

### 4.3. Fluorescence Real-Time qPCR

The RNA sequencing samples that were isolated were also used to perform real-time quantitative (qPCR) analysis. Total RNA samples with 2 μg per reaction were reverse-transcribed into cDNA using M-MLV Reverse Transcriptase (Promega, Madison, WI, USA). qPCR-specific primers were designed based on the candidate gene sequences close to the 3’ end using Beacon Designer 7.0 software from Premier Biosoft International, Palo Alto, CA, USA. The *Histone3* (GenBank NO: AF024716) was used as reference gene. The primers of *histone3* and *GarWRKY5* were forward primer 5’-CGGTGGTGTGAAGAAGCCTCAT-3’ and reverse primer 5’-AATTTCACGAACAAGCCTCTGGAA-3’ and forward primer 5’-GCCTTGTCATTTCATGGTGGA-3’ and reverse primer 5’-GGGTTGTCGTTGCCTTGC-3’, respectively. The light cycler carried out using IQ SYBR Green Supermix (Bio-Rad, Hercules, CA, USA) based on the manufacturer’s instructions and the qPCR products were quantified using ABI 7500 Fast (Applied Biosystems, Foster City, CA, USA). The amplification reaction conditions for PCR were performed as follows: 94 °C for 3 min, followed by 40 cycles at 94 °C for 15 s, 60 °C for 15 s, and 72 °C for 30 s. The relative expression levels were calculated using the 2^−ΔΔCt^ method with three biological replicates and three experimental replicates [42].

### 4.4. Analysis of Salt Tolerance in Transgenic Arabidopsis Plants

For the salt tolerance of *GarWRKY5* transgenic *Arabidopsis* plants during the seed germination stage, 50 seeds of T_2_ generation transgenic lines (three lines for *GarWRKY5*) were surface-sterilized and sown on Murashige and Skoog (MS) medium with and without 150 mM NaCl. The WT was used as control. After 10 days, the germination rate of the seeds was calculated. At least three biological replicates were carried out in the experiment. To further verify that overexpression of *GarWRKY5* could enhance tolerance to salt stress during vegetative growth, sterilized seeds of WT and T_2_ transgenic *Arabidopsis* were sown in soil. After 20 days, the seedlings were grown in a pot supplemented with 150 mL NaCl solution (150 mM/L), and distilled deionized water was used as control. The phenotype of the seedlings was observed after four weeks. For the determination of antioxidant enzyme activity, three-week-old seedlings from WT and T_2_ generation of three *GarWRKY5*-overexpressing transgenic lines (*GarWRKY5*-1, *GarWRKY5*-6, and *GarWRKY5*-14) were soaked in 150 mM/L NaCl solution for 24 h. Leaves of at least 10 seedlings were collected from the WT and three transgenic lines. The activity of POD and SOD was determined based on the procedure described by Liu et al. (2008) [43]. The amount of enzyme required to cause 50% inhibition of nitro blue tetrazolium (NBT) reduction was considered as one unit of SOD activity. The SOD was measured at 560 nm by an ultraviolet spectrophotometer. The activity of POD was analyzed at 470 nm using guaiacol as a substrate by the ultraviolet spectrophotometer. The experiment was performed in 50 mmol/L phosphate buffer, 50 mmol/L guaiacol, and 2% H_2_O_2_, and 2 μl of enzyme extract was added. The data was recorded after adding 2.0 ml 20% chloroacetic acid. All the above procedures of enzyme extraction were carried out at 0–4 °C. The enzyme assays were performed in three biological replicates.

### 4.5. Virus-Induced Gene Silencing Assays

In order to knockdown the expression of the *GhWRKY5* gene, a 389 bp fragment of the *GhWRKY5* cDNA from TM-1 was amplified using the VIGS primers. The resulting PCR product with double digestion (XbaI and KpnI) was recombined into XbaI–KpnI-digested pTRV2 in order to produce pTRV2::*GhWRKY5*. The pTRV2::*GhWRKY5* vector was introduced into the *Agrobacterium* strain GV3101 by means of electroporation (Bio-Rad, Hercules, CA, USA). For the VIGS assay, the GV3101 containing pTRV1, pTRV2 (mock-treated controls), pTRV2::*GhWRKY5*, and pTRV2::*GhCLA1* were used. The strains were grown overnight at 28 °C with shaking at 150 rpm in Luria–Bertani (LB) broth containing two antibiotics—kanamycin and rifampicin—in concentrations of 50 mg/L each. The *Agrobacterium* were harvested by centrifugation for 5 min at 5000 rpm and resuspended in infiltration buffer (10 mM MES, 10 mM MgCl_2_, and 200 mM acetosyringone) to a final OD_600_ of 2.0. The *Agrobacterium* strains with the TRV1 or TRV2 vectors were mixed by equal volume and incubated for three hours at 28 °C. Seedlings with mature cotyledons but no visible true leaf (about one week postemergence) were infiltrated by inserting the *Agrobacterium* suspension into the cotyledons surface by lightly pricking with a syringe. The plants were grown at 23 °C (day/night) in an illumination incubator with a 16 h light/8 h dark cycle and at a relative humidity of 60% for one week [44]. For the identification of *GhWRKY5*, a cotton elongation factor gene (*GhEF1α*) was used as the internal control in RT-PCR. Primers for *GhWRKY5* and *GhEF1α* were forward primer 5’-AGCAAACATGTCTTGGAAC-3’ and reverse primer 5’-CGGCCTTTCAAAACTGA-3’ and forward primer 5’-AGACCACCAAGTACTACTGCAC-3’ and reverse primer 5’-CCACCAATCTTGTACACATCC-3’, respectively.

VIGS experiments were repeated at least three times with more than five plants for each construct per repeat.

### 4.6. Transcriptome Sequencing and DEG Analysis

Approximately, a total of 8 μg RNA per sample was used. The RNA purity and quality were assessed using an Agilent 2100 Bioanalyzer (Agilent Technologies, Palo Alto, CA, USA) and a Qubit^®^ 2.0 Fluorometer (Invitrogen, Carlsbad, CA, USA). Three biological replicate RNA samples were prepared for library construction and sequencing. Based on the manufacturer’s protocol of kit (Illumina^®^ TruSeq^™^ RNA Sample Preparation Kit (Illumina Inc. San Diego, CA, USA)), the cDNA libraries were prepared. The cDNA libraries were sequenced based on Illumina HiSeq™ 2000 with 100 bp single end reads each. The basis of gene expression analysis was as follows: the number of unambiguous clean tags for each gene was calculated and then normalized to TPM (number of transcripts per million clean tags) [45,46].

The reads from RNA-Seq were aligned to the reference genome (TAIR10 data) using Tophat version 2.0.11, which was compatible with Bowtie2 version 2.2.1 [47]. All reads were allowed only one nucleotide mismatch. Clean reads mapping to reference sequences from multiple genes were filtered out. For DEG analysis, we adopted a conservative criterion by choosing consistent results of cuffdiff (ref), with |log_2_ (fold change) | ≥ 1 and significant expression with false discovery rate (FDR) of <0.05 and gene fragments per kilobase million (FPKM) value of ≥1.

### 4.7. Availability of Data and Material

All raw transcriptomics reads have been deposited in NCBI Sequence Read Archive (http://www.ncbi.nlm.nih.gov/sra). The BioProject and SRA accession are PRJNA529955.

## 5. Conclusions

Based on the data presented in this study, we hypothesize that *GarWRKY5* may be a positive transcription regulator in plant response to high salinity stress through the ROS-scavenging system, such as activating expression of GST and SOD genes by the jasmonic acid- or salicylic acid-mediated signaling pathway.

## Figures and Tables

**Table 1:** The 19 differentially expressed genes (DEGs) involved in salt stress and osmotic stress processes based on Gene Ontology (GO) enrichment analysis.

Gene ID	Gene Annotation	log_2_^(OE 0 d vs. WT 0 d)^	Padj	W-Box
**AT1G02920**	glutathione S-transferase F7	1.50	4.71E-15	6
**AT1G02930**	glutathione S-transferase F6	1.25	1.15E-20	8
**AT1G08830**	superoxide dismutase [Cu-Zn]	1.16	9.40E-31	6
**AT1G27730**	zinc finger protein STZ/ZAT10	1.19	4.30E-07	5
**AT1G43160**	ethylene-responsive transcription factor RAP2-6	1.59	8.06E-08	8
**AT1G52400**	beta glucosidase 18	1.51	2.56E-18	3
**AT1G56650**	transcription factor MYB75	2.50	9.70E-37	3
**AT1G65690**	late embryogenesis abundant (LEA) hydroxyproline-rich glycoprotein	1.29	6.27E-06	7
**AT2G28190**	copper/zinc superoxide dismutase 2	1.44	5.12E-56	10
**AT2G33380**	caleosin 3	1.63	1.28E-33	4
**AT2G38750**	annexin D4	1.29	3.93E-23	4
**AT2G38760**	annexin D3	1.25	1.62E-12	6
**AT3G16470**	JA-responsive protein 1	1.07	6.09E-12	4
**AT3G49580**	protein RESPONSE TO LOW SULFUR 1	1.41	3.80E-06	5
**AT4G23600**	cystine lyase CORI3	1.06	4.06E-04	5
**AT4G30650**	putative low temperature and salt responsive protein	1.19	6.06E-18	10
**AT5G24660**	protein RESPONSE TO LOW SULFUR 2	1.80	2.40E-10	6
**AT5G24770**	acid phosphatase VSP2	1.31	1.21E-06	5
**AT5G59820**	high light responsive zinc finger protein ZAT12	1.24	1.42E-05	8

## Supplementary Materials

Supplementary materials can be found at https://www.mdpi.com/1422-0067/20/21/5244/s1.

## Abbreviations

DEGs	differentially expressed genes
GO	Gene Ontology
POD	peroxidase
SOD	superoxide dismutase
TF	transcription factor
VIGS	virus-induced gene silencing
