# Supplementary figures and images for: Transcription Factor GarWRKY5 Is Involved in Salt Stress Response in Diploid Cotton Species (Gossypium aridum L.)
Source: Int J Mol Sci. 2019 Oct 23;20(21):5244. doi: 10.3390/ijms20215244 (PMC6862181; doi:10.3390/ijms20215244)

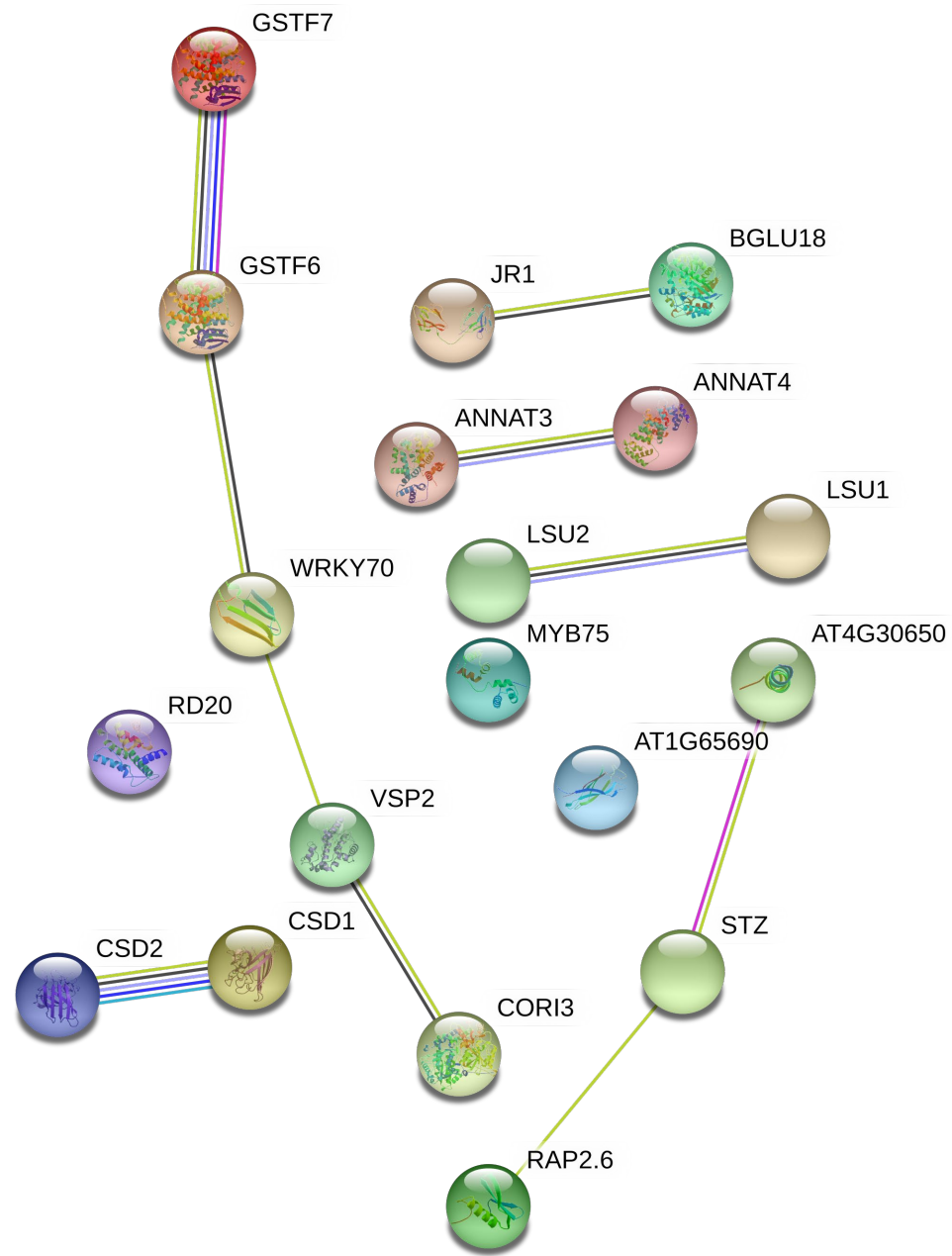

Supplement: Supplementary file 1 [file ijms-20-05244-s001.zip › Fig S1 prediction of function protein association network.pdf]
